# HEALTH, SOCIAL ISOLATION, AND 12-YEAR MORTALITY ACROSS DIFFERENT COMMUNITY-BASED SENIOR HOUSING TYPES

**DOI:** 10.1093/geroni/igae098.3849

**Published:** 2024-12-31

**Authors:** Seoyeon Ahn, Sojung Park, Eunsun Kwon, BoRin Kim

**Affiliations:** Duke-NUS Medical School, Singapore, Singapore; Washington University in St. Louis, St. Louis, Missouri, United States; Washington University in St. Louis, St. Louis, Missouri, United States; University of New Hampshire, Durham, New Hampshire, United States

## Abstract

The literature on subsidized senior housing for low-income older adults primarily focuses on affordability and health outcomes like mortality. In contrast, research on other senior housing models often emphasizes the social environment, creating a disconnect that may lead to misunderstandings about factors influencing health and well-being across different housing types. This study examines the influence of socio-economic status, health conditions, and social isolation on 12-year mortality across various community-based senior housing types. Data were drawn from twelve waves of the National Health and Aging Trend Study (2011-2022), involving 6,534 community-dwelling adults (obs=29,929) aged 70 or older who died during the study period. Housing types were categorized into five groups: 1) subsidized senior housing (SSH), 2) senior housing with low-income status and housing cost burden (HCB), 3) senior housing without low-income status and non-HCB, 4) traditional homes with low-income status or HCB, and 5) traditional homes without low-income status and non-HCB. A hierarchical discrete-time event history model using logistic regression was employed to assess the influence of health conditions, social isolation, and housing type on mortality risk. We found that (1) low income or HCB significantly increased mortality risk across all housing types; (2) chronic health conditions and severe social isolation were strong predictors of higher mortality; and (3) socially isolated individuals had a lower mortality risk when living in senior housing compared to traditional homes. These findings highlight the importance of housing affordability policies and suggest that congregate senior housing offers protective benefits for socially vulnerable older adults.

